# A systematic quality rating of available mobile health apps for borderline personality disorder

**DOI:** 10.1186/s40479-022-00186-w

**Published:** 2022-06-01

**Authors:** Lena Sophia Steubl, Josephin Reimann, Laura Simon, Yannik Terhorst, Michael Stach, Harald Baumeister, Lasse Bosse Sander, Eva-Maria Messner

**Affiliations:** 1 Institute of Psychology and Education, Department of Clinical Psychology and Psychotherapy, Ulm University, Ulm, Germany; 2 Institute of Databases and Information Systems (DBIS), Ulm University, Ulm, Germany; 3 Institute of Psychology, Department of Rehabilitation Psychology and Psychotherapy, Albert-Ludwigs-University of Freiburg, Freiburg, Germany

**Keywords:** BPD, Internet- and mobile-based interventions, IMIs, Treatment, Quality rating

## Abstract

**Background:**

Mobile health apps (MHAs) may offer a mean to overcome treatment barriers in Borderline Personality Disorder (BPD) mental health care. However, MHAs for BPD on the market lack transparency and quality assessment.

**Methods:**

European app stores were systematically searched, and two independent trained reviewers extracted relevant MHAs. Employed methods and privacy and security details documentation of included MHAs were extracted. MHAs were then assessed and rated using the German version of the standardized Mobile Application Rating Scale (MARS-G). Mean values and standard deviations of all subscales (engagement, functionality, aesthetics, information, and therapeutic gain) and correlations with user ratings were calculated.

**Results:**

Of 2977 identified MHAs, 16 were included, showing average quality across the four main subscales (*M* = 3.25, *SD* = 0.68). Shortcomings were observed with regard to engagement (*M* = 2.87, *SD* = 0.99), potential therapeutic gain (*M* = 2.67, *SD* = 0.83), existing evidence base (25.0% of included MHAs were tested empirically), and documented privacy and security details. No significant correlations were found between user ratings and the overall total score of the MARS-G or MARS-G main subscales.

**Conclusions:**

Available MHAs for BPD vary in quality and evidence on their efficacy, effectiveness, and possible adverse events is scarce. More substantial efforts to ensure the quality of MHAs available for patients and a focus on transparency, particularly regarding privacy and security documentation, are necessary.

**Supplementary Information:**

The online version contains supplementary material available at 10.1186/s40479-022-00186-w.

## Background

Borderline Personality Disorder (BPD) is the most common personality disorder in clinical settings, with a prevalence rate of up to 20% among psychiatric inpatients [1]. The prevalence rate in the general population is estimated to be 1-2% [1]. While there is a wide range of effective treatment approaches (e.g., dialectical behavior therapy, DBT; mentalization-based therapy, MBT; [2]), only a limited number of those affected receive evidenced-based treatment [3]. This may partly occur due to a lack of trained mental health care professionals and limited budgets [4, 5].

Compared to traditional face-to-face settings, Internet- and mobile-based interventions (IMIs) offer various advantages that might help overcome potential treatment barriers (e.g., scalability, anonymity, flexibility; [6, 7]). Mobile health apps (MHAs), in particular, offer users the opportunity to flexibly integrate treatment into their daily lives, given their status as ubiquitous devices. Consequently, it may be argued that the treatment gap for BPD could be reduced with the help of MHAs that are already available.

Several meta-analyses showed the efficacy of IMIs for various mental disorders (e.g., [8–10]), as yet mostly based on Internet-based interventions, while evidence for MHAs is less established [11]. Correspondingly, a recent scoping review on technology-based interventions for BPD shows that while evidence for Internet-based interventions is emerging, there is only little evidence for MHAs for BPD [12]. However, results on all five included MHAs supported their acceptance and/or usability [12]. A recent systematic review and meta-analysis on seven randomized controlled trials (RCTs) of MHAs for adults with common BPD symptoms [13] showed no significant effects between conditions with and without (e.g., waitlist, treatment-as-usual) MHAs on BPD-related symptoms. Yet, the number of included studies was rather small, only half of the MHAs included in the systematic review and meta-analysis were commercially available, and there is a wide range of MHAs on the market not tested empirically [13].

Regarding MHAs available on the market, prior research has shown that user ratings are strongly influenced by user-friendliness and are therefore often misleading regarding clinical app quality, while they may be the only source of information on the quality for potential users (e.g., [14–16]). This is especially important considering the fact that the use of MHAs can also come along with risks and side effects (e.g., insufficient data protection, treatment without informed consent, critically wrong information, inadequate handling of crises [17]).

Therefore, the objective of this study is to investigate MHAs for BPD already available on the market and thereby go beyond previous reviews on published efficacy and effectiveness trials. The aim is to (a) examine the content and evidence of MHAs for BPD and (b) assess the quality of the MHAs for BPD with regard to engagement, functionality, aesthetics, information, and potential therapeutic gain.

## Methods

### Search strategy

MHAs available in German and British app stores (i.e., Apple App Store for iOS MHAs and Google Play Store for Android MHAs) were systematically searched with BPD-related English, German, and French search terms (see Additional file 1) employing a web crawler [18]. The validity of this procedure has been proven in previous studies (e.g., [19, 20]). All terms were searched individually as logical operations and truncation cannot be used in the app stores. The search was conducted in September 2020.

### Inclusion and exclusion criteria

The inclusion process was divided into two parts and performed by two reviewers with a degree in psychology (JR, JW) under the supervision of a researcher with a Master’s degree in psychology and in training to become a licensed psychotherapist (LSS). First, all identified MHAs were screened, whether their title and description indicated that the MHA (a) was developed for BPD, (b) its description specifically refers to BPD (no general skill training), (c) aims at those affected by BPD or their relatives, and (d) met no other exclusion criteria (i.e., only indicated in blended care settings, wearables or other accessories necessary). The latter exclusion criteria were applied to ensure comparability and detailed and comprehensive assessments of included MHAs. Duplicates were excluded automatically and manually. Second, all MHAs screened as eligible (both free and with costs) were downloaded and assessed for further information. The MHAs were downloaded and installed either on a Huawei P10 lite (model WAS-LX1A) or an Apple iPhone 6 s (model NN0X2ZD/A). MHAs were included for the rating and analysis if (a) they worked to the degree that assessment was possible and (b) the provided content addressed BPD interventionally (e.g., psychoeducation, skills training).

### Assessment of included apps

Pairs of two independent reviewers with a degree in psychology (KB, JW, JR) conducted the classification and quality rating for all included apps. They were supervised by a researcher with a Master’s degree in psychology and in training to become a licensed psychotherapist (LSS), who also solved classification and rating conflicts. In case MHAs were available for different operating systems, the MHAs were rated independently. The assessment was performed using the German version (MARS-G; [21]) of the Mobile Application Rating Scale (MARS; [22]).

#### General characteristics

An adapted version of the classification page of the MARS-G was used to examine MHA characteristics. The following information was assessed: (a) name, (b) platform, (c) developer, (d) store link, (e) yearly price in Euro (if available in other currencies, the price was converted), (f) affiliation (i.e., unknown, commercial, university, non-governmental organization, government), (g) user star rating and the number of ratings (only user ratings with a minimum number of three ratings were included), (h) existence of a disclaimer in the MHA description that the MHA does not replace psychotherapeutic treatment/diagnostics, (i) employed methods (i.e., psychoeducation, data collection, feedback, reminders, skills training, mindfulness exercises, breathing exercises, relaxation exercises, body exercises, goal tracking), and (j) privacy and security details documentation (i.e., password protection, informed consent, contact details, login, emergency functions, safety measurements for mobile loss). Conflicts were defined as any disagreement.

#### Quality rating

The multidimensional MARS(−G) quality rating includes 19 items on four different main subscales, which are evaluated on a five-point Likert scale (1 = inadequate, 2 = poor, 3 = acceptable, 4 = good, and 5 = excellent): (a) engagement with five items (entertainment, interest, customization, interactivity, target group), (b) functionality with four items (performance, ease of use, navigation, gestural design), (c) aesthetics with three items (layout, graphics, visual appeal), and (d) information with seven items (accuracy of MHA description, goals, quality of information, the quantity of information, visual information, credibility, evidence base; [21, 22]). The MARS has excellent internal consistency (Cronbach’s alpha = .90) and high levels of inter-rater reliability (IRR; two-way mixed *ICC* = .79; 95% CI [.79, .83]; [22, 23]). Internal consistencies of the four main subscales are also very high (α = .80-89, Median = .85) and inter-rater reliabilities are fair to excellent (*ICC* = .50-.80, Median = .65; [22, 23]). Correspondingly, the MARS-G shows acceptable to excellent internal consistency for all subscales (ω = .74-.91) as well as for the overall score (ω = .81, 95% CI [.74, .86]; [21]). The correlations of the corresponding dimensions of the MARS and MARS-G range from *r* = .93-.98 [21].

Furthermore, the potential therapeutic gain was assessed as an additional subscale in accordance with the MARS-G with four items (gain for patients, gain for therapists, risks and side effects, ease of implementation into routine healthcare; [21]).

The rating was performed by two independent and trained reviewers.

### Data analysis

Data analysis was conducted in R [24]. For all following calculations, the ratings of the two raters were averaged. Mean values and standard deviations were calculated for each subscale and across all four main subscales (engagement, functionality, aesthetics, information). In addition, the correlations between the user rating and the mean values of each subscale and the mean value across all four main subscales were calculated. For quality assurance, inter-rater reliability between the two raters was examined by intraclass correlation based on a two-way model with absolute agreement. Hereby, an ICC below .50 is suggested to describe poor agreement, between .50 and .75 moderate agreement, between .75 and .90 good agreement, and above .90 excellent agreement [25].

## Results

### Search

The systematic search identified 2977 MHAs, of which 12 distinct MHAs with a total of 16 versions (0.5%) were eligible for inclusion. Of these, 11 (68.8%) were developed for the Android operating system and five (31.3%) for iOS. Three MHAs (Emoteo, DBT Coach, Simple DBT Skills Diary Card) were available for both operating systems. The process of inclusion is detailed in the flow chart (Additional file 2).

Annual prices of included MHAs ranged from 0 to 125.98€, 12 MHAs (75.0%) were free of initial costs. However, four of the latter (33.3%) offered in-app purchases. The majority of MHAs (*n* = 12; 75.0%) came from a commercial source, followed by MHAs from universities (*n* = 2; 12.5%). One MHA each (6.3%) came from an NGO and an unknown source. User ratings ranged from 3.0 to 5.0 stars (*M* = 4.1; *SD* = .62). The app store descriptions of the majority of included MHAs (*n* = 9; 56.3%) did not include a disclaimer about the non-replacement of psychotherapeutic treatment/diagnostics even though treatment guidelines do not suggest MHAs as a first-line approach [26]. Detailed characteristics are displayed in Additional file 3.

With regard to employed methods, all included MHAs contained psychoeducational content. Most MHAs (*n* = 13; 81.3%) collected data (e.g., in terms of monitoring, tracking). Moreover, eight MHAs (50.0%) contained mindfulness exercises, six MHAs (37.5%) each contained breathing exercises and feedback, five MHAs (31.3%) skills training, four (25.0%) relaxation exercises, and three (18.8%) reminders. None of the included MHAs contained body exercises or goal tracking. A detailed listing of employed methods can be found in Table 1.

**Table 1:** Employed methods in included MHAs in descending order of the total quality mean score

Name	Platform	Background	Psycho-education	Data collection	Feedback	Reminder	Skills training	Mindfulness exercises	Breathing Exercises	Relaxation Exercises	Body Exercises	Goal Tracking
DBT Coach	iOS	DBT	✓	✓		✓		✓	✓			
Skills2Go für Borderliner	iOS	DBT	✓	✓			✓	✓		✓		
DBT Coach	Android	DBT	✓	✓	✓	✓	✓	✓	✓			
DBT Travel Guide	Android	DBT	✓	✓	✓		✓	✓	✓	✓		
Simple DBT Skills Diary Card	Android	DBT	✓	✓			✓	✓				
Simple DBT Skills Diary Card	iOS	DBT	✓	✓		✓	✓	✓				
Borderline Personality D Test	iOS	–	✓	✓	✓							
Psychopedia	Android	–	✓	✓	✓							
Borderline Explained the truth about BPD	Android	–	✓									
Emoteo	iOS	DBT	✓	✓				✓	✓	✓		
Emoteo	Android	DBT	✓	✓				✓	✓	✓		
Borderline-Persönlichkeitsstörung	Android	–	✓									
Borderline Explained Premium	Android	–	✓	✓	✓							
PD Test - Persönlichkeitsstörungstest	Android	–	✓	✓	✓				✓			
Personality Disorder Test	Android	–	✓	✓								
Borderline Personality Disorder; Causes, Treatment	Android	–	✓									

With regard to privacy and security details documentation, the majority of the included MHAs (*n* = 15; 93.8%) provided contact details, and seven MHAs (43.8%) offered password protection. Five MHAs (31.3%) contained emergency functions, four (25.0%) included informed consent, and three (18.8%) required a login. Detailed privacy and security information for each included MHA is displayed in Table 2.

**Table 2:** Security and privacy details documentation of included MHAs in descending order of the total quality mean score

Name	Platform	Password Protection	Informed Consent	Contact Details	Login	Emergency Functions	Safety Measurements for Mobile Loss
DBT Coach	iOS	✓		✓	✓	✓	
Skills2Go für Borderliner	iOS			✓			
DBT Coach	Android	✓	✓	✓	✓	✓	
DBT Travel Guide	Android	✓		✓		✓	
Simple DBT Skills Diary Card	Android	✓	✓	✓			
Simple DBT Skills Diary Card	iOS	✓	✓	✓	✓		
Borderline Personality D Test	iOS						
Psychopedia	Android			✓			
Borderline Explained the truth about BPD	Android			✓			
Emoteo	iOS	✓		✓		✓	
Emoteo	Android	✓		✓		✓	
Borderline-Persönlichkeitsstörung	Android			✓			
Borderline Explained Premium	Android			✓			
PD Test - Persönlichkeitsstörungstest	Android		✓	✓			
Personality Disorder Test	Android			✓			
Borderline Personality Disorder; Causes, Treatment	Android			✓			

Four of the included MHAs (25.0%) were tested empirically (“DBT Coach” and “Emoteo”, both available for Android and iOS). Two uncontrolled pilot studies on “DBT Coach” (*n* = 22; *n* = 16) showed decreased emotion intensity, urges to self-harm and substance use, depression, and distress related to the MHA and good acceptability and usability when used as an adjunct to treatment [27, 28]. One uncontrolled pilot study on “Emoteo” as an adjunct to treatment with 16 participants showed high satisfaction with the MHA and a significant decrease in aversive tension [29]. None randomized controlled clinical trial was identified for any of the included MHAs.

### Quality rating

Overall, the quality of included MHAs over the four main subscales was average (*M* = 3.25, *SD* = 0.68). Functionality was the highest-rated subscale (*M* = 3.78, *SD* = 0.55), followed by information (*M* = 3.35, *SD* = 0.69), aesthetics (*M* = 3.00, *SD* = 0.83), and engagement (*M* = 2.87, *SD* = 0.99). Results from the MARS-G quality rating are displayed in Table 3.

**Table 3:** Means of the four main subscales of the MARS-G (Messner et al., 2019) ratings in descending order of the total mean score (range: 1 to 5)

Name	Platform	Engagement	Functionality	Aesthetics	Information	Overall Quality
DBT Coach	iOS	4.4	4.8	5	4.1	**4.6**
Skills2Go für Borderliner	iOS	4.2	4.8	4	4.3	**4.3**
DBT Coach	Android	4.3	3.6	4.3	4	**4.1**
DBT Travel Guide	Android	3.6	4.1	3.7	4.2	**3.9**
Simple DBT Skills Diary Card	Android	3.9	3.9	3	3.8	**3.7**
Simple DBT Skills Diary Card	iOS	3.7	3.5	2.5	3.7	**3.3**
Borderline Personality D Test	iOS	2.3	4.2	2.8	3.3	**3.2**
Psychopedia	Android	1.9	4	3	3.3	**3.1**
Borderline Explained the truth about BPD	Android	2.2	4.1	2.7	3.1	**3**
Emoteo	iOS	2.9	3	2.7	3.4	**3**
Borderline-Persönlichkeitsstörung	Android	2.1	3.9	2.8	2.7	**2.9**
Borderline Explained Premium	Android	2.1	3.9	2.3	3.2	**2.9**
Emoteo	Android	2.7	3.2	2.5	3.3	**2.9**
PD Test - Persönlichkeitsstörungstest	Android	1.9	3.1	2.2	2.8	**2.5**
Personality Disorder Test	Android	2.2	3	2.2	2.5	**2.5**
Borderline Personality Disorder; Causes, Treatment	Android	1.5	3.4	2.3	1.8	**2.2**

Inter-rater reliability on item level across all four main subscales was good (*ICC* = .90; 95% CI [.87, .92]) and reliabilities for each of the subscales separately ranged from good to excellent (*ICC* = .75-.94).

No significant bivariate correlations were found between the user ratings and the overall total score of the MARS-G (*r*(11) = .26, 95% CI [−.34, .71]; *p* > .05) or MARS-G main subscales (*r*(11) = .05-.32, *p* > .05).

**Fig. 1:**
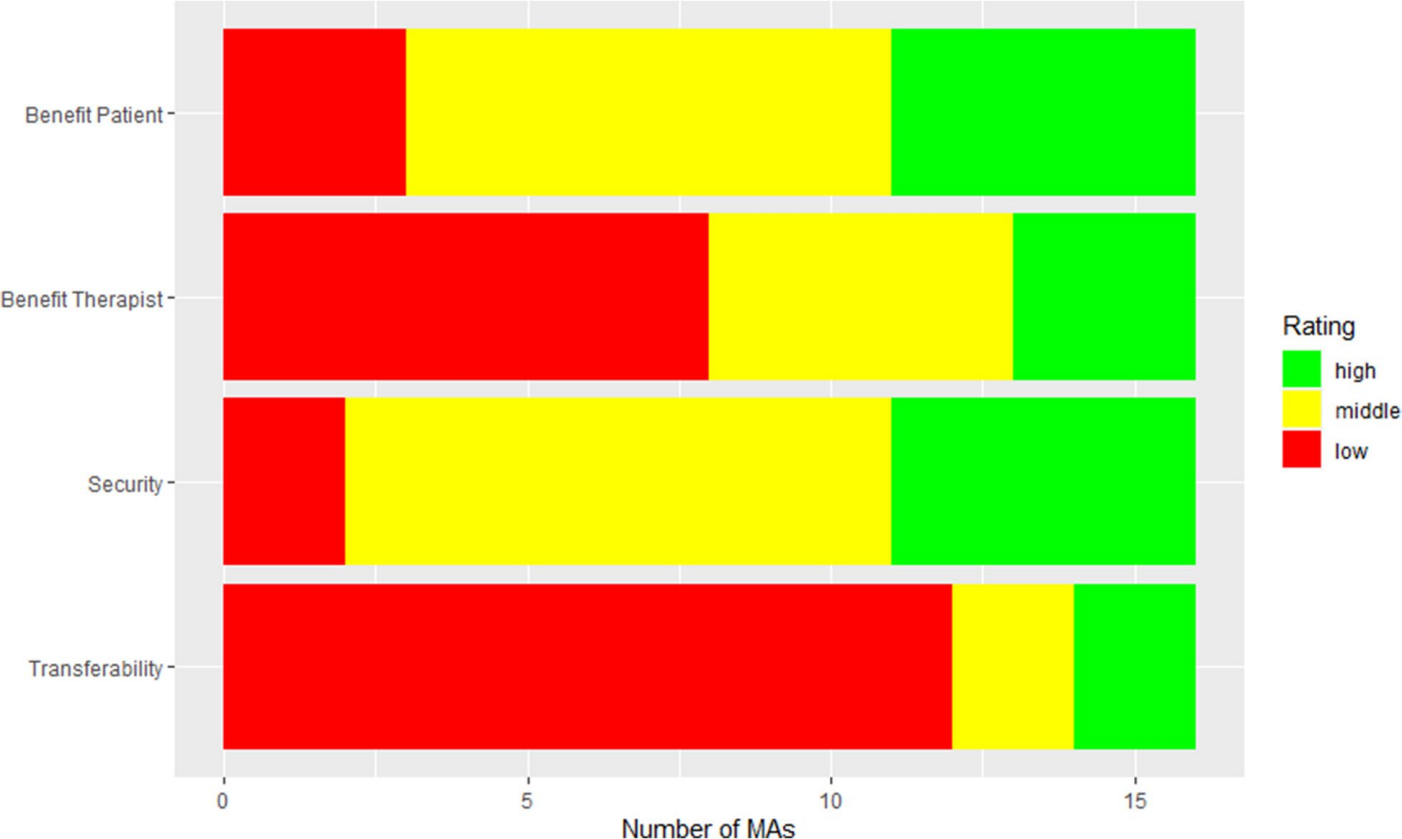
Ratings on the subscale therapeutic gain across all included MHAs. The item risk was renamed to security to clarify encoding directions. Ratings lower than 2.5 were categorized as low, ratings between 2.5 and 4 as middle, and ratings of 4 and higher as high

The additional subscale on potential therapeutic gain showed lower ratings (*M* = 2.67, *SD* = 0.83). Therapeutic gain ratings per item across all apps are displayed in Fig. 1.

## Discussion

The present study summarizes the findings of a systematic search and quality rating of MHAs for BPD in German and British app stores (Apple App Store for iOS MHAs and Google Play Store for Android MHAs). The 16 included MHAs showed varying quality with particular shortcomings in engagement, existing evidence base, privacy and security details documentation, and potential therapeutic gain. This is in line with previous studies on MHAs for other mental health issues (e.g., [16, 30–34]). Of note, previous results on the validity of user ratings (e.g., [14–16]) could also be reproduced, with no correlations between these ratings and both the overall quality of included MHAs and individual subscales.

Lack of engagement facilitating components seems to be a recurring problem in MHAs for mental health disorders (e.g., [16]), which points out the contradiction between the unique possibilities MHAs offer in terms of persuasive design (e.g., personalization, real-time feedback [35, 36]; and the low adherence to MHAs in real-world treatment [37]. In contrast, the subscale information, arguably the most important subscale from a therapeutic perspective, showed a moderate quality of the included MHAs. However, the large range from 1.8 to 4.3 reflects the existence of both MHAs with almost all relevant information provided in a concise and accurate manner and MHAs that do not provide suitable information at all.

While we were able to identify three studies on the acceptability, usability, and effectiveness of four of the included MHAs (25.0%), none of them was a randomized controlled trial, highlighting the limited evidence base. This may be due to the fast-moving nature of the field of MHAs and the fact that they are scientifically developed and evaluated. MHAs are often not made publicly available outside related studies [13]. However, it points also toward the need for more research on actually available MHAs employing rigorous study designs to gain knowledge on the effectiveness of available MHAs for BPD. Of note, in Germany, there are efforts to establish a billing model by which scientifically evaluated MHAs can be prescribed by health care providers. However, the extent of the scientific evaluation remains debated [38].

Whereas shortcomings in the area of privacy (e.g., in terms of insufficient data protection, treatment without informed consent) may be equally relevant for all MHAs, security issues (e.g., lack of emergency functions) may be particularly important in the treatment of BPD, given patients’ self-harm tendencies, frequent crises, and suicidal behaviors [3]. However, only five (31.2%) of the included MHAs offered any such feature. Of note, this builds a rather low threshold for privacy and data security, given that the present study examined these domains only on content and documentation level by means of the MARS rating scale, not a technological level (e.g., threat and vulnerability analysis, data handling). Hence, only 31% of all examined MHAs overcoming this basic threshold can be viewed as alarming regarding patient safety in the context of uncontrolled use of MHAs for BPD.

Observed shortcomings on the subscale of potential therapeutic are also in line with concerns regarding the fit of stand-alone MHAs for BPD patients. More precisely, it is assumed that the need for interpersonal contact as an important mechanism of treatment in this population cannot be met in self-help IMIs [3]. Quite to the contrary, interpersonal contact in face-to-face therapy is regarded as an essential context and corrective experience for dealing with conflicts and intensive emotions [3]. This opinion of (face-to-face) interpersonal contact and therapeutic relationship being a necessary precondition for psychotherapeutic approaches to work is not new to the field of digital mental health care [39, 40]. However, evidence of the last two decades has highlighted that therapeutic relationship can be established digitally, being probably not as important as assumed, at least in a digital treatment context and represent no necessary precondition for mental disorder treatments to work [39, 40]. Still, this general statement needs to be examined and validated for each mental disorder separately and might be particularly challenged in the context of personality disorders. In this context, the recent systematic review and meta-analysis on seven RCTs on two stand-alone MHAs and three MHAs offered as an adjunct to other interventions for adults with common BPD symptoms showed no significant effect compared to control conditions (i.e., placebo, treatment-as-usual, waitlist, anger management group that was available for both intervention and control group) on symptoms (Hedges’ *g* = − 0.07, 95% CI [−.25, .12]) and general psychopathology (Hedges’ *g* = 0.30, 95% CI [− 0.14, 0.75]; [13]). Thus, MHAs might fall short, and the lack of interpersonal contact or therapeutic guidance [41] could be one reason for them being not effective to date. Of note, though most included studies in this systematic review and meta-analysis did not report any serious adverse events, many had strict inclusion and exclusion criteria (e.g., regarding suicidal ideation or severe depression). Assuming that effects in the more complex BDP population not included in the studies are even smaller and adverse events may be more common, this further limits the recommendation of publicly available MHAs for BPD.

However, while the evidence on the effectiveness of particularly stand-alone MHAs for BPD is scarce, well developed and safe MHAs may still be beneficial if integrated into a concept of blended psychotherapy, i.e., the combination of online intervention elements with standard psychotherapeutic care [3, 42–46]. Blended therapy may provide the possibility to combine both the need for and safety of interpersonal contact in the treatment of BPD and the advantages of IMIs and MHAs in particular and offers several advantages beyond that (e.g., patient empowerment, therapist support by standardized materials; [46–50]).

## Limitations

When interpreting the results of this study, there are some important limitations that must be considered. First, the search results may be limited by the selected search strings and the language restriction (English, German, and French). While the present search strategy was quite extensive in the number of different terms, nonetheless, future studies could expand the present findings by including other languages. Second, MHAs indicated in blended care settings or MHAs using wearables, or other accessories were excluded, which might have further limited the number of included MHAs. Third, MHAs might have been missed due to the restriction to the German and British Google Play and Apple App Store due to the limitations of the web crawler used. However, as Google Play and Apple App Store together hold a market share of around 99% [51], the loss of relevant MHAs should be negligible. Yet, it cannot be guaranteed that the findings of this study are transferable to the respective MHA versions in other stores or that included MHAs are also available in other major markets (e.g., United States, India, China). Fourth, the availability of MHAs in stores changes rapidly, and the present study must be understood as a snapshot at the time of the search. Fifth, privacy and data security details documentation was only assessed on a descriptive level. In line with the present findings, a recent study on privacy and data security in MHAs for depression and smoking cessation showed inadequate or insufficient information in present privacy policies [52]. Therefore, a closer look at the privacy policies of MHAs may be worthwhile in future studies. Ultimately, however, MHAs need to be tested regarding data security and privacy by means of, e.g., thread analysis and Medical Device Regulation testing [53–55]. Sixth, there was only a limited number of MHAs included, which decreases the power of correlation analyses. Consequently, non-significant correlations between user ratings and MHA quality should be interpreted with caution.

## Implications

﻿Despite these limitations, this study has several strengths, including the extensive search strategy and the standardized rating by two independent reviewers that allow an overview of available MHAs for BPD and initial conclusions. More precisely, regarding the usage of MHAs in the treatment of BPD, results point to the need for further studies on the efficacy, effectiveness and possible adverse events of MHAs for BPD and independent information platforms (e.g., Mobile Health App Database: http://mhad.science; MIND: https://mindapps.org/) to provide reliable information on the quality and content of MHAs. Ultimately, only the combination of a thorough content, system and evidence testing can inform about the quality and thus recommendability of MHAs for BPD.

## Conclusion

Our systematic rating of 16 MHAs for BPD indicated an average overall quality following the standardized MARS-G quality rating. Future MHAs should focus more on the unique technical aspects of smartphones to enhance engagement and pay more attention to privacy and security details. Evidence on the efficacy, effectiveness and possible adverse events of MHAs for BPD included in this systematic rating is limited, preventing reliable recommendations regarding their usage. However, some of the available MHAs offer content that may help to close the treatment gap, at least in the blended therapy context.

## Supplementary Information

**Additional file 1.**

**Additional file 2.**

**Additional file 3.**

## Data Availability

The datasets used and analyzed during the current study are available from the corresponding author on reasonable request. Data will only be shared for scientific purposes. Data sharing agreements may have to be signed depending on the request. Support is depending on current resources.

## Abbreviations

BPD: Borderline Personality Disorder
DBT: Dialectical behavior therapy
IMI: Internet- and mobile-based intervention
MHA: Mobile health application
MARS: Mobile Application Rating Scale (Stoyanov et al., 2015)
MARS-G: Mobile Application Rating Scale – German version (Messner et al., 2020)
MBT: Mentalization-based therapy
MHAD: Mobile Health App Database
RCT: Randomized controlled trial
