# Supplementary material for: A systematic quality rating of available mobile health apps for borderline personality disorder
Source: Borderline Personal Disord Emot Dysregul. 2022 Jun 1;9:17. doi: 10.1186/s40479-022-00186-w (PMC9158356; doi:10.1186/s40479-022-00186-w)
Supplement: Supplementary file 1 — Additional file 1. [file 40479_2022_186_MOESM1_ESM.docx]

**Search String**

| **German** | **English** | **French** |
| --- | --- | --- |
| BPS | BPD | TPB |
|  | EUPD | TPL |
| Borderline | borderline |  |
| Borderline Persönlichkeit | borderline personality | personnalité borderline |
| emotional instabile Persönlichkeitsstörung | emotionally unstable personality disorder | trouble limite de la personnalité |
| Borderline Störung | borderline disorder | trouble borderline |
|  |  | trouble limite |
| Borderline Therapie | borderline therapy | thérapie borderline |
|  |  | thérapie limite |
| Borderline Syndrom | borderline syndrome |  |
| DBT | DBT | TCD |
| DBT Borderline | DBT borderline |  |
| dialektisch-behaviorale Therapie | dialectical behavior therapy | thérapie comportementale dialectique |
| MBT | MBT | TBM |
| MBT Borderline | MBT borderline | TBM borderline |
| mentalisierungsbasierte Psychotherapie | mentalization-based therapy | thérapie sur la base de mentalisation |
|  | mentalisation-based therapy |  |
| Fertigkeiten Borderline | skills borderline | aptitudes borderline |
| DBT Fertigkeitentraining | DBT skills training | entrainment aptitudes TCD |
| Trauma Borderline | trauma borderline | traumatisme borderline |
| Impulsivität | impulsivity | impulsivité |
| Instabil Borderline | unstable borderline | instable borderline |
| Emotion Borderline | emotion borderline | émotion borderline |
| emotional-instabil |  | émotionnel-instable |
| Emotionsregulation | emotion regulation | régulation des émotions |
| emotionale Krisen | emotional crises | crises émotionnelles |
| Krisenplan Borderline | crisis plan borderline | plan de crise borderline |
| Tracking Borderline | self-monitoring borderline | autosurveillance borderline |
| Stimmung Borderline | mood borderline | Humeur borderline |
| Stimmung BPS | mood BPD | humeur TPB |
| Ritzen Hilfe | cutting help | couper aide |
| selbstverletzendes Verhalten | self-harming behavior | comportements autodestructeurs |
|  | self-harming behaviour |  |
| Selbstverletzung | self harm | automutilation |
|  | self injury |  |
| Selbstverletzung Borderline | self harm borderline | automutilation borderline |
| Selbstmordgedanken | suicidal thoughts | pensées suicidaires |
