# Supplementary figures and images for: A systematic quality rating of available mobile health apps for borderline personality disorder
Source: Borderline Personal Disord Emot Dysregul. 2022 Jun 1;9:17. doi: 10.1186/s40479-022-00186-w (PMC9158356; doi:10.1186/s40479-022-00186-w)

**Flow chart**

**
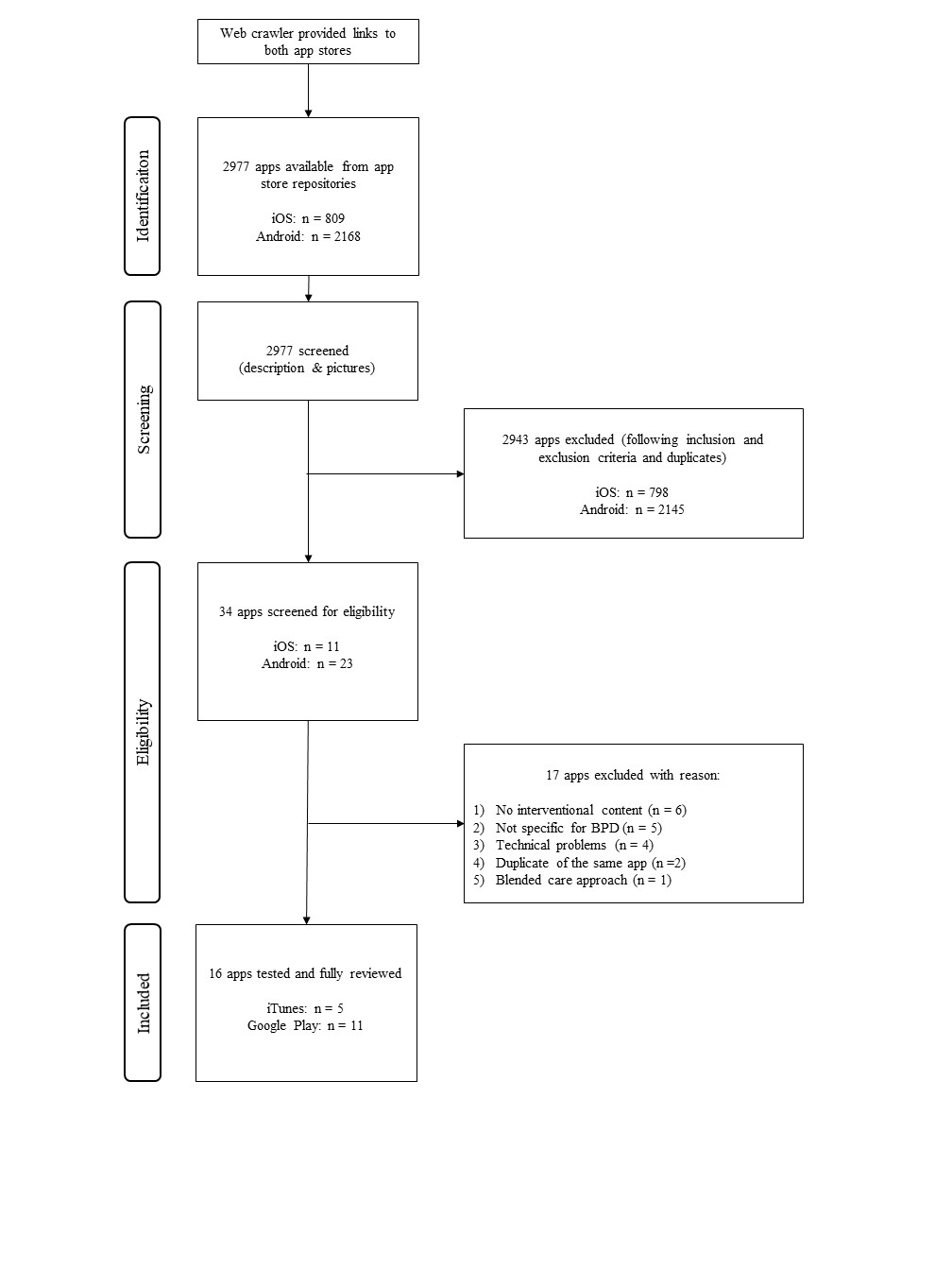
**

Supplement: Supplementary file 2 — Additional file 2. [file 40479_2022_186_MOESM2_ESM.docx]
