# Supplementary material for: A systematic quality rating of available mobile health apps for borderline personality disorder
Source: Borderline Personal Disord Emot Dysregul. 2022 Jun 1;9:17. doi: 10.1186/s40479-022-00186-w (PMC9158356; doi:10.1186/s40479-022-00186-w)
Supplement: Supplementary file 3 — Additional file 3. [file 40479_2022_186_MOESM3_ESM.docx]

**Characteristics of included mobile health apps.**

| **Name** | **Platform** | **Developer** | **Price (yearly)** | **Affiliation** | **User Rating (ratings total)^a^** | **Disclaimer in app description (no replacement for psychotherapeutic treatment/ diagnostics)** | **Link** | **Description** |
| --- | --- | --- | --- | --- | --- | --- | --- | --- |
| DBT Coach | iOS | Swasth Inc. | 0 €^b^ | Commercial | 4.7 (3) | Yes | https://apps.apple.com/de/app/dbt-coach/id1452264969#?platform=iphone | - video lessons and animations to learn and practice DBT skills - exercises and homework - crisis survival list - community tools - diary card for skills and target behaviours - assessment tools to check progress - integrates with a clinician app |
| Skills2Go for Borderliner | iOS | woopla GmbH | 0 €^b^ | Commercial | 4.4 (67) | Yes | <https://apps.apple.com/de/app/skills2go-f%C3%BCr-borderliner/id1013569339> | - daily mood diary and analysis - skills training (e.g., mindfulness, relaxation) |
| DBT Coach | Android | Swasth Inc. | 0 €^b^ | Commercial | 3.8 (1.053) | No | <https://play.google.com/store/apps/details?id=co.swasth.dbtcoach> | - video lessons and animations to learn and practice DBT skills - exercises and homework - crisis survival list - community tools - diary card for skills and target behaviours - assessment tools to check progress - integrates with a clinician app |
| DBT Travel Guide | Android | Dialexis Advies | 0 € | Commercial | 4.2 (105) | No | https://play.google.com/store/apps/details?id=com.dialexis.dgtcoach&hl=en&gl=US | - tools to regulate emotions and control impulses - information about DBT and BPD - skills and mindfulness exercises - crisis section - practical guidelines for DBT therapist - digital diary card and agreements between client and therapist |
| Simple DBT Skills Diary Card | Android | Pop! Pop! | 0 €^b^ | Commercial | 3.0 (59) | No | <https://play.google.com/store/apps/details?id=poppop.AndroidDBTSkills&hl=de&gl=US> | - daily diary card - information - description and examples of skills - print format of data |
| Simple DBT Skills Diary Card | iOS | POP POP LLC | 0 €^b^ | Commercial |  | No | <https://apps.apple.com/de/app/simple-dbt-skills-diary-card/id666921665#?platform=iphone> | - daily diary card - information - description and examples of skills - print format of data |
| Borderline Personality D Test | iOS | PocketShrink | 2.27 € | Commercial |  | No | https://apps.apple.com/gb/app/borderline-personality-d-test/id640824582 | - self-diagnosis tool - interactive forum and support group |
| Psychopedia | Android | Metanoia Foundation | 0 € | NGO | 4.7 (19) | No | <https://play.google.com/store/apps/details?id=com.app.metanoia> | - assessment - information |
| Borderline Explained the truth about BPD | Android | Psycnet Software | 0 € | Commercial | 4.5 (356) | No | <https://play.google.com/store/apps/details?id=com.andromo.dev859449.app1007337&hl=en_US&gl=US> | - information |
| Emoteo | iOS | HUG Hôpitaux universitaires de Genève | 0 € | University | 5.0 (4) | Yes | <https://apps.apple.com/ch/app/emoteo/id961175516> | - tools and exercises |
| Emoteo | Android | HUG Hôpitaux universitaires de Genève | 0 € | University | 3.0 (37) | Yes | https://play.google.com/store/apps/details?id=ch.hug_ge.emoteo | - tools and exercises |
| Borderline-Persönlichkeits-störung | Android | lahcenappsinc | 0 € | Commercial | 3.7 (7) | No | <https://play.google.com/store/apps/details?id=com.lahcenappsinc.borderlinepersonalitydisorder> | - information |
| Borderline Explained Premium | Android | Psycnet Software | 1.99 € | Commercial |  | No | <https://play.google.com/store/apps/details?id=net.newandromo.dev7998.app636121> | - information |
| PD Test – Persönlichkeits-störungstest | Android | People ‘n Droid | 0 €^b^ | Commercial | 3.9 (482) | Yes | <https://play.google.com/store/apps/details?id=it.ppndrd.disturbidellapersonalita&hl=de&gl=US> | - assessment |
| Personality Disorder Test | Android | Psycnet Software | 0 € | Commercial | (90) | Yes | <https://play.google.com/store/apps/details?id=com.newandromo.dev7998.app175700> | - information on personality disorders - assessment |
| Borderline Personality Disorder; Causes, Treatment | Android | Health Info | 0 € | Unknown | 3.5 (17) | Yes | <https://play.google.com/store/apps/details?id=com.healthinfo.borderlinepersonalitydisorder.cause.prevention.management.treatment.diagnosis> | - information |

^a^reviews were documented 10.02.2021; only ratings with at least 3 ratings are reported ^b^in-app purchases available
